# Leveraging Student-Athlete Mental Health Through an AI-Augmented Mobile Platform: The ThriveNudge Study Protocol

**DOI:** 10.3390/bs16020268

**Published:** 2026-02-11

**Authors:** Sameer Chakraborty, Nicholas Mendro, Longxi Li

**Affiliations:** 1 Paul G. Allen School of Computer Science and Engineering, University of Washington, Seattle, WA 98105, USA; sdc28@uw.edu; 2 Arlington High School, Arlington, WA 98223, USA; mendrn@uw.edu; 3 Center for Leadership in Athletics, College of Education, University of Washington, Seattle, WA 98105, USA

**Keywords:** mental health, youth sport, physical activity, large language models, mHealth, coach education

## Abstract

Playing sports remains one of the most common avenues for youth engagement in physical activity. Yet mental health challenges, such as performance anxiety, depressive symptoms, reduced motivation, and burnout, place many young athletes at risk. As key mediators of sport participation, coaches’ roles are often underscored in recognizing shifts in athlete motivation, behavior, or well-being. Gaining better insight into athlete mental health status may enable coaches to provide timely support and strengthen athlete and team well-being. In this study protocol, we employ a mixed-methods design, evaluating the effectiveness of an AI-augmented mobile application (i.e., ThriveNudge) in promoting the mental health of youth athletes. ThriveNudge helps coaches monitor athlete mental health, flag mood disruptions, and practice supportive communication via simulated chats. A target sample of four interscholastic teams (with athletes aged 14–18 years) and their head coaches will be recruited. Teams will be cluster-randomized to either the intervention condition (*n* = 2), receiving pre-season training to implement ThriveNudge, or to a waitlist control condition (*n* = 2). Primary outcomes, including athlete burnout, motivation, coach–athlete relationships, and sport enjoyment, will be measured using psychometric scales administered online. Semi-structured interviews will be conducted with coaches and athletes in the experimental group to collect qualitative data on user interface and user experience. We hypothesize that teams using ThriveNudge will report lower athlete anxiety and burnout, higher intrinsic motivation and enjoyment, and stronger coach–athlete relationships than athletes in control teams. We aim to provide a scalable and accessible digital platform that safeguards youth mental health.

## 1. Introduction

A growing body of research has established the youth mental health crisis as a pressing public health concern (Shim et al., 2022). Between the ages of 10 and 25, young people undergo rapid biological, social, and emotional changes (McGorry et al., 2024). During this developmental period, the onset of common mental health disorders, including anxiety and depression, represents a major contributor to the global burden of disease in youth (Swann et al., 2018). In the United States, the prevalence of identified mental health conditions has increased, with 40% of children meeting criteria by age 18 (Shim et al., 2022). Rising mental ill-being has also been linked to premature mortality, including suicide and physical illness, among young people (McGorry et al., 2024). Youth sport participation, meanwhile, has been associated with formative experiences during this transformative period of development (Gould, 2016). Yet the current sport landscape has not fully leveraged technological advances, such as artificial intelligence (AI), to support sport as a scalable mechanism for reducing youth mental health burden. In the current study, we propose a protocol to test the efficacy of ThriveNudge (hereafter TN), an AI-augmented digital platform designed to safeguard youth athlete mental health. TN is a non-commercial, research-developed mobile app prototype that facilitates coach–athlete communication and helps coaches foster autonomy-supportive interactions.

Sport-based approaches such as TN build on the idea that well-being can be supported through resources embedded in youths’ everyday settings. Youth sport programs can foster psychosocial skill development (Freeman & Rees, 2009), and the social climate of these settings can shape young people’s satisfaction of basic psychological needs (Vallerand, 2000). When appropriately designed, sport environments can support youth social and emotional well-being by strengthening positive relationships and self-esteem (Swann et al., 2018). Accordingly, research has consistently linked adolescent sport participation with higher levels of life satisfaction (Guddal et al., 2019). Among high school students, physical activity has been associated with better mental health and lower psychological distress (Hamer et al., 2009). Further, research shows that youth who build skills to sustain self-esteem and cultivate positive relationships are better positioned to carry positive outcomes of sport participation from childhood into young adulthood (Tahira, 2023).

The extent to which sport supports youth well-being often depends on the environment, particularly the relational context created by coaches. In youth sport, the quality of the coach–athlete relationship is positively associated with athletes’ need satisfaction and well-being (Felton & Jowett, 2013; Li et al., 2025). Research indicates the importance of a team environment characterized by trust, support, and autonomy in meeting athletes’ developmental needs (Coussens et al., 2025). When young athletes perceive low levels of care and support, they may be more likely to report stress, burnout, reduced motivation, and negative peer interactions (Gould, 2016). In contrast, coaches can help cultivate caring, autonomy-supportive climates through regular check-ins that support athletes’ basic psychological needs (Jowett, 2017).

Recent technological advances also present an underutilized opportunity to strengthen coach–athlete relationships and support athlete well-being. Many existing tools primarily facilitate logistics, such as schedule alerts and administrative updates. Other platforms, including AthleteTalk (AthleteTalk, 2025) and Dailyhuman (Dailyhuman, 2025), enable athletes to track performance, access strategies for physical or emotional resilience, and monitor aspects of mental health. Yet comparatively few solutions are designed to support coaches directly by building their communication skills, strengthening relationships, and embedding athlete well-being into everyday interactions within sport settings.

TN is an app designed to strengthen coach–athlete relationships by supporting a sport climate that meets youth athletes’ basic psychological needs. Development was informed by Self-Determination Theory (SDT), with features that support intrinsic motivation (Ryan & Deci, 2000, 2017). The design premise is that need-supportive communication in sport is central to young athletes’ growth, development, and well-being (Felton & Jowett, 2013). In TN, athletes complete brief daily well-being reflections that are summarized for coaches as scaled check-in indicators and can be viewed across coach-selected time windows. Coaches also interact with integrated large language models (LLMs) that simulate conversations with athletes experiencing varying levels of anxiety and motivation, allowing coaches to rehearse responses and receive prompts aligned with autonomy-supportive communication. These components are intended to strengthen coaches’ need-supportive behaviors and improve athletes’ perceptions of relationship quality and support, which have been associated with greater satisfaction of competence and relatedness needs (Felton & Jowett, 2013). Perceived support has also been linked to positive health outcomes, including stronger coping in stressful situations and lower depressive symptoms (Freeman & Rees, 2009).

Meanwhile, emerging evidence suggests that AI-enabled simulations can support skill development and training outcomes across applied settings. For example, one study used AI-generated simulated patients to train students in clinical interviewing, highlighting the advantages of scalable delivery and opportunities for repeated practice in training contexts (García-Torres et al., 2025).

The purpose of the present study protocol is to preliminarily evaluate the effectiveness of TN as an AI-augmented tool intended to support youth athletes’ well-being. The protocol also examines implementation in an interscholastic setting, with attention to need-supportive communication, coach–athlete relationship quality, and student-athletes’ well-being and sport experiences over the course of a season. Given the pilot nature of the study and the limited number of clusters, analyses will emphasize descriptive and estimation-focused approaches rather than confirmatory hypothesis testing. Exploratory research questions (RQ_1–4_) are listed below:

RQ_1_: What patterns of change across the season are observed in student-athletes’ sport satisfaction, intrinsic motivation, mental well-being, and burnout among TN teams compared with waitlist control teams?

RQ_2_: How do student-athletes’ perceptions of autonomy-supportive coaching behaviors vary over time between TN teams and waitlist control teams?

RQ_3_: What patterns of change in coach–athlete relationship quality are observed across the season between TN teams and waitlist control teams?

RQ_4_: Among teams using TN, how is variation in coach engagement with the platform (e.g., login frequency and simulation use) associated with observed trends in student-athlete outcomes?

## 2. Materials and Methods

### 2.1. Setting and Design

This study will be conducted in collaboration with two high schools in North America, with data collected from two interscholastic teams at each high school. Two selected high schools will be randomly assigned to either the intervention group, which will use TN, or to a waitlist control group, which will not receive the intervention during the study period. Both groups will follow the rules set by their state’s interscholastic governing body and have access to standard school resources. Coaches will be recruited and informed consent will be obtained prior to student-athlete recruitment. Then, their athletes will be recruited accordingly. Informed consent will be obtained from parents or legal guardians before student-athlete recruitment, and assent will be obtained from all student-athletes. The interventional effect will be evaluated using a quasi-experimental design, and student-athlete outcomes will be measured at the beginning, midpoint, and end of the sport season. To gain an understanding of the experience of using TN, a subgroup of coaches and student-athletes will be recruited for interviews with open-ended questions. Altogether, we will analyze the qualitative and quantitative data using statistical and thematic analyses accordingly. While no monetary incentives will be provided, coaches and student-athletes will be invited to a brief end-of-season presentation where the research team will share key findings and practical takeaways. Participants will also receive a printed brochure with evidence-informed, actionable strategies and resources on mental health and team well-being. This protocol has been preregistered on the Open Science Framework (Registration DOI: 10.17605/OSF.IO/85S9G).

### 2.2. Participants

The study cohort will include high school student-athletes from two public schools, with two teams recruited from each school. Inclusion criteria are (1) full-time enrollment in the selected high schools; (2) on an interscholastic team; (3) age between 14 and 18 years at the beginning of the study; (4) fluency in English; and (5) access to a mobile device (iOS or Android). Head coaches of each participating team will also be recruited.

### 2.3. Sample Size Calculation

The present study is not powered for any confirmatory inference. The targeted roster sizes (approximately 15–25 athletes per team) are sufficient to evaluate feasibility and engagement and to provide preliminary clustering estimates (i.e., intraclass correlation coefficient). We will report these estimates with 95% confidence intervals. Precision will be limited by the small number of teams. Published intraclass correlation coefficient (ICC) values will also inform power for a future cluster-randomized trial.

### 2.4. Procedure

Coaches and high school student-athletes will be recruited from a purposive sample of two public high schools within a district that reflects a diverse demographic composition. Schools will serve as the unit of randomization and will be randomly assigned to either the TN intervention group or the waitlist control group. Four weeks prior to the start of the season, head coaches will be contacted via email, using the contact information provided by the district athletic director. Following pre-screening to confirm their coaching role, coaches will be invited to complete an online enrollment survey that includes the purpose of the study, its procedures, and an option to opt out. Coaches who consent to participate will be listed under the intervention or control condition. Two teams from each school will then be randomly selected for inclusion, resulting in a total of four participating teams. To prevent potential contamination, both teams from the same school will remain in the same condition, consistent with school-level group assignment. Teams in the intervention group will have access to TN throughout the season, whereas teams in the control group will serve as a waitlist control and receive access after the study is completed.

Parental consent and student-athlete assent will follow IRB-approved procedures appropriate for research with minors. Given that the study involves a non-clinical, educational mobile platform and poses no greater than minimal risk, the research team will seek for a passive (opt-out) parental consent process, consistent with school district research policies.

Parents or legal guardians will receive an informational letter via email two weeks before data collection, describing the study purpose, procedures, confidentiality protections, and voluntary nature of participation. The letter will include both an online opt-out form and contact information for the research team. Those who do not opt out will be considered to have provided consent. Student-athletes will then provide electronic assents prior to participation, with the option to withdraw at any time without penalty.

If more than 50% of parents or guardians on a selected team decline participation, that team will be replaced by another eligible team from the same school, which will inherit the school’s randomized condition to preserve the integrity of the cluster randomization. No re-randomization will occur at the team level. Parents or guardians of student-athletes in the waitlist control condition will receive similar study information but will be informed that their child(ren) will complete online surveys across the season and gain access to the TN mobile application after the season ends.

For the intervention group, a 30 min online pre-season training will be offered to orient participants to TN, including installation, initial setup, and core functions. Both the intervention and control groups will complete online surveys at three time points: the beginning of the season (baseline), mid-season, and the end of the season. Before each survey, participants will be reminded that their participation is voluntary, that they may withdraw at any time without penalty, and that their responses will be anonymous and not shared with coaches, parents, or school staff.

All survey data will be de-identified and stored on encrypted servers, accessible only to the research team. Coaches will not have access to individual student responses, only to aggregated, anonymized team-level summaries where appropriate. Neither personal-level data will be visible to schools nor to the TN developers.

Within two weeks of the postseason, coaches and student-athletes in the control group will be introduced to TN through a short tutorial and guided installation instructions via email. The study protocol and all related materials will be reviewed and approved by the Institutional Review Board prior to participant recruitment. All procedures will be conducted in accordance with the ethical principles outlined in the Declaration of Helsinki.

### 2.5. Participant Support and Safety

While TN was not developed for clinical or diagnostic purposes, participant safety and mental well-being will be prioritized throughout the study. The platform includes a “Resources” page in both coach and student-athlete versions, linking users to publicly available mental health support materials, national helplines, and school-based counselors. A medical disclaimer is displayed upon login, stating that the platform does not provide crisis counseling or clinical assessment.

During pre-season orientation, the research team will review support and safety procedures with coaches and student-athletes. Participants will be informed that their app use and survey responses are confidential and not monitored in real time. Coaches will not have access to individual-level data or well-being responses and will not “track” specific student-athletes’ moods or app activity. Instead, any optional check-in features within TN are anonymized and used only for aggregate research analyses conducted by the research team.

A tiered escalation protocol will be in place for any concerning responses that emerge in the study’s online surveys (e.g., endorsement of severe distress, self-harm intent, or perceived safety threats):

1. Automated flagging: Surveys containing keywords or responses suggesting imminent risk will automatically generate a confidential alert to the principal investigator. Automated flagging is triggered by the presence of predefined keywords and phrases commonly associated with acute psychological distress or self-harm risk. Examples include references to suicidal ideation (e.g., “I want to kill myself,” “I don’t want to be here anymore”), self-harm behaviors (e.g., “cutting,” “hurting myself”), or imminent safety concerns (e.g., “I plan to hurt myself today”). These examples are illustrative rather than exhaustive. Flagging serves solely as an initial screening mechanism to prompt human review and does not initiate any automated decision-making or intervention.
2. Review: The investigator will review flagged responses within 24 h to determine whether escalation is warranted.
3. Referral and documentation: If a credible concern for participant safety is identified, the investigator will contact the school counselor to initiate a standard welfare check, following district safety procedures. No information will be shared with coaches or peers. When escalation is deemed necessary, only the minimum information required to support participant safety is shared with the designated school counselor. This information is limited to the nature of the concern (e.g., indication of severe distress or self-harm risk), the participant’s school affiliation, and the timing of the flagged response. No raw survey responses, application usage logs, or simulated conversation content will be shared. All communications follow established district welfare-check procedures, and identifying information will be disclosed only to authorized school liaison directly responsible for student support.
4. Record keeping: All flagged cases and follow-up actions will be documented in a secure file accessible only to the principal investigator. Records will be deleted four weeks after the study concludes.

This protocol ensures that any participant identified as potentially at risk is connected promptly to appropriate, school-based mental health support while maintaining confidentiality and adhering to ethical standards. All personnel involved in data collection will complete human subjects research ethics training prior to study initiation.

### 2.6. Coach and Student-Athlete Interviews

To complement the quantitative data, brief semi-structured interviews will be conducted at the end of the season with a purposive subsample of coaches (*n* = 2) and student-athletes (*n* = 6) to gather exploratory, usability-focused feedback on the TN platform’s interface, acceptability, and perceived impact on team culture and well-being. An interview guide with open-ended questions developed by the research team will be used to elicit user experiences, perceived benefits and challenges, and recommendations for future iterations.

Interviews will be conducted in person or via Zoom, depending on participants’ preferences. With consent, interviews will be audio-recorded, transcribed verbatim, de-identified, and reviewed for transcription accuracy. Participation in interviews will be voluntary. Confidentiality will be protected by using pseudonyms in transcripts and reports. No identifying information will be shared with schools. Participants may withdraw at any time without penalty.

Given the pilot nature of the study and its emphasis on feasibility, acceptability, and user experience, a small number of interviews are deemed appropriate to identify salient interface- and experience-related issues that may inform future refinement of the platform. Student-athletes will be selected for interviews using purposive sampling to capture a range of perspectives based on variation in sport type, self-reported engagement with the application, and observed patterns of app use over the season.

### 2.7. App-Based Intervention Development

TN was developed as a cross-platform mobile tool using React Native version 0.83 with Expo for the frontend, enabling deployment across iOS devices and other platforms. The backend was implemented in Python 3 using Flask, connected to a Firebase Firestore database for secure storage of anonymized user data, including session logs and survey completion records. The application delivers simulated coach–athlete conversations, powered by LLMs, and hosted via external Application Programming Interface (APIs). Each simulation presents the participant with a dynamic student-athlete persona characterized by varying motivational styles, challenges, and attitudes. Simulated student-athlete responses vary based on selected settings. See examples of two Simchat dialogues below:

(1) *Coach* (*human*): “Hey, how are you feeling lately?”
  *Athlete* (*AI*): “I’ve been stressing out a lot about the upcoming game. Just hoping everything goes smoothly.”
  *Coach* (*human*): “You’re doing great. I have full confidence in you. Can’t wait to see what you do this weekend.”
  *Athlete* (*AI*): “I know you believe in me, but I just can’t shake this nervousness. What if I mess up in front of everyone? Coach, can we talk about some strategies to calm down before the game? To be honest, I’m really worried.”

(2) *Coach* (*human*): “Hey, how are your feeling lately?”
  *Athlete (AI):* “I’ve been okay, just trying to stay focused on the games. Got a big match coming up, so I’m trying to manage my nerves.”
  *Coach* (*human*)*:* “I appreciate your focus and dedication to our team. Is there anything I can do to help calm your nerves?”
  *Athlete* (*AI*): “I’ve been practicing deep breathing exercises, helps a bit. Just need to stay positive and believe in the team.”

From an implementation perspective, TN is deployed as a modular client–server system designed for scalability and minimal data exposure. The mobile client, built with React Native and Expo, handles user interaction, local state management, and on-device preprocessing (including lightweight redaction). A Python-based Flask backend manages authentication, session orchestration, and secure communication with external services. Anonymized metadata and usage logs are stored in Firebase Firestore, while simulated conversation responses are generated via external LLM APIs using standardized, non-personal prompt templates. This separation of concerns allows core application logic, data storage, and language-model inference to operate independently, reducing system complexity and supporting secure deployment in school-based settings.

#### 2.7.1. Coach-Facing Module: Features, Rationale, and Validity

The coach version of TN provides a practice environment where coaches engage in simulated student-athlete dialogues. Guided by user-centered design, the system prompts coaches to practice motivational interviewing (MI) skills, such as open-ended questions, affirmations, and reflective listening, which have been shown to strengthen athlete motivation and well-being in applied sport settings (Miller & Rollnick, 2013; Hagger & Chatzisarantis, 2007). Simulations are brief, lasting 5–8 min, to encourage repeated practice and reduce cognitive load while maintaining ecological relevance to the lived experiences of high-school athletes.

To ensure training validity, we incorporated fidelity checks based on the Motivational Interviewing Treatment Integrity (MITI) 4.0 coding system, a standardized and psychometrically tested framework for evaluating MI proficiency (Moyers et al., 2016; Pierson et al., 2007). This provides structured feedback on the quality and consistency of coaches’ use of MI-aligned communication strategies.

We also integrated sentiment analysis modules that identify emotional cues, such as frustration, optimism, or disengagement, in simulated athlete responses. This draws on validated work in natural language processing and mental health, where AI models and sentiment tools have been shown to detect depression, anxiety, and stress markers in text (Cosco et al., 2019; Miner et al., 2016; Shatte et al., 2019). Importantly, our module is designed not as a diagnostic tool but as a reflective aid: after each simulation, coaches receive feedback linking their communication strategies to how an athlete might perceive motivation or psychological safety.

Finally, psychological safety is emphasized because evidence from sport and organizational psychology shows it is critical for athlete well-being, learning, and team functioning (Walton et al., 2023; Newman et al., 2017). By combining MI fidelity metrics, sentiment-based reflective feedback, and structured debriefs, TN provides coaches with an evidence-grounded training tool to enhance supportive communication and autonomy-supportive coaching practices. To evaluate coaches’ communication output within simulations, we triangulate across (1) process fidelity, assessed via MITI coding of coach dialogue transcripts (Moyers et al., 2016; Pierson et al., 2007); and (2) alignment with athlete-facing outcomes, interpreted through validated instruments such as the Warwick-Edinburgh Mental Well-Being Scale (WEMWBS) (Tennant et al., 2007), the Sport Satisfaction Instrument (SSI) (Granero-Gallegos et al., 2014), and the Coach–Athlete Relationship Questionnaire (CART-Q) (Jowett, 2009; Woolliams et al., 2021). Using these established psychometric frameworks to code and interpret coach outputs strengthens construct validity and ensures that training effects can be meaningfully compared to prior work in sport psychology and digital mental health interventions (Miner et al., 2016; Newman et al., 2017; Prior et al., 2024; Shatte et al., 2019; Walton et al., 2023). Coaches can view individual athlete well-being scores in a secure dashboard that lists de-identified athlete IDs. This feature is designed to help coaches monitor patterns of motivation or distress and provide timely support. However, the graphical interface primarily presents aggregated data, such as team-level averages and variability, over adjustable time windows (e.g., 7-, 14-, or 30-day rolling summaries). Individual-level data are visible only as numerical entries without personal identifiers.

#### 2.7.2. Athlete-Facing Module: Features, Rationale, and Validity

Additionally, the student-athlete version emphasizes well-being and coping support. Student-athletes engage with brief simulated conversations designed to help them explore coping strategies, set goals, and reflect on challenges through guided prompts. Real-time sentiment analysis is applied to adjust the trajectory of the simulation. For example, if a simulated student-athlete expresses discouragement, the model shifts toward generating nudges that validate their feelings and provide motivational support, whereas expressions of confidence prompt reinforcement-oriented nudges. To ensure safety and complement professional support, the app also includes a resources page that links to public mental health resources, school counseling services, and crisis hotlines.

**Figure 1:**
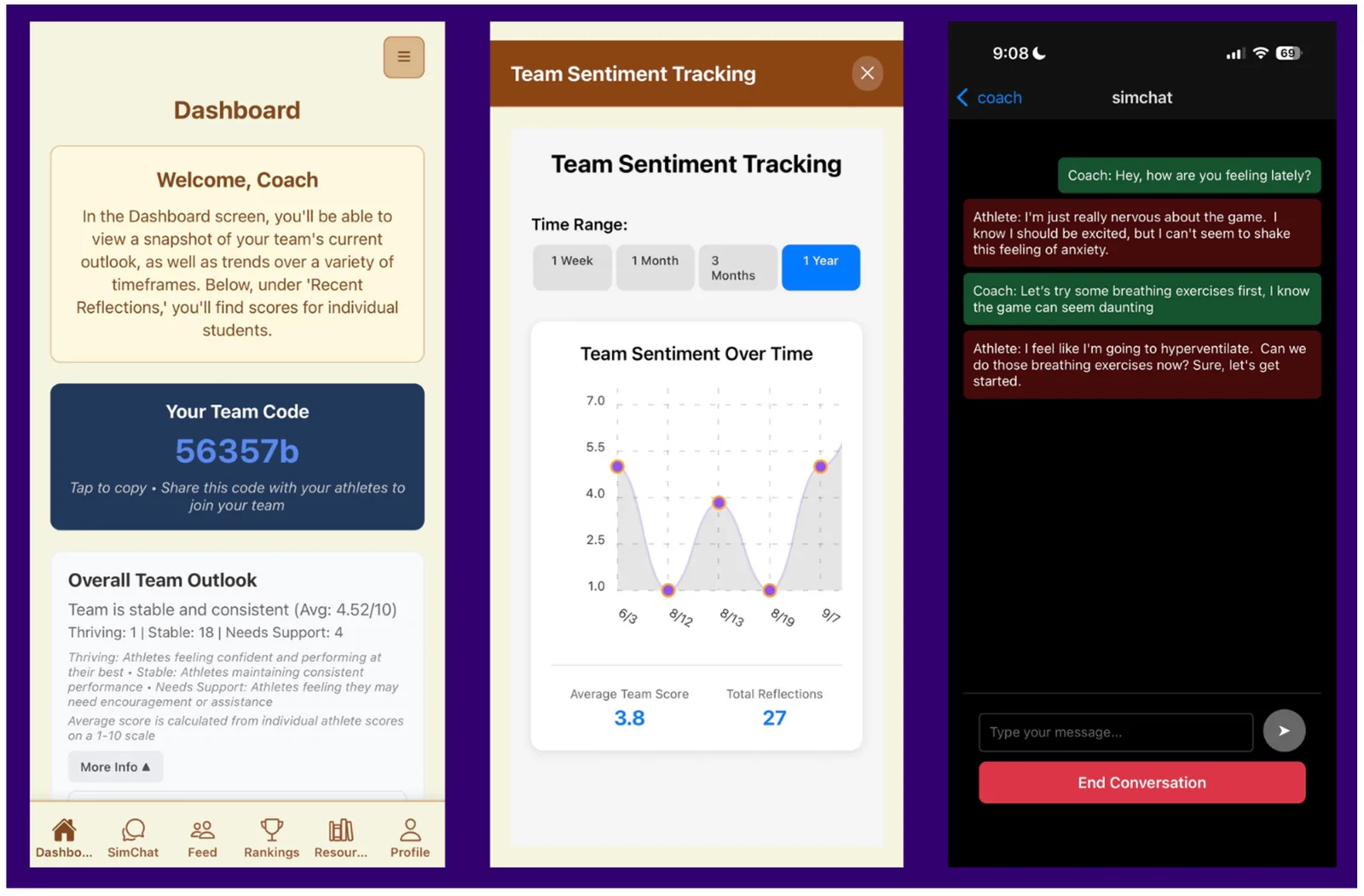
Core features and user interface design for coaches.

**Figure 2:**
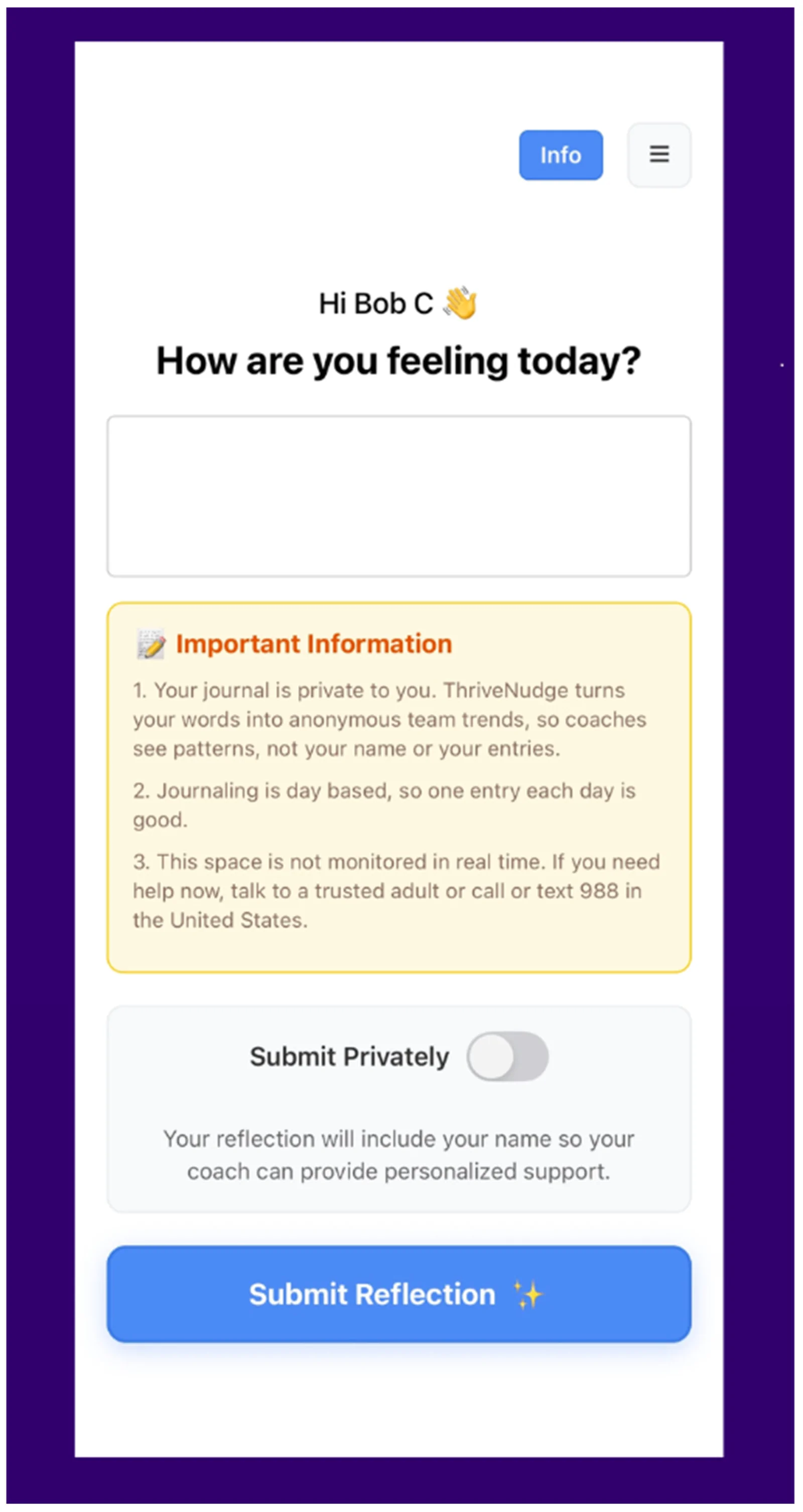
Core feature and user interface design for student-athletes.

Data safety, privacy, and ethical considerations are central to TN design. All user data is anonymized before storage, and no personally identifiable information is collected. Conversations are logged in a de-identified form for research and evaluation purposes, and participants are informed that TN serves as a training and support tool rather than a diagnostic or therapeutic service. Core features of TN are illustrated in Figure 1 and Figure 2.

#### 2.7.3. Data Protection & LLM Vendor Risk

All data management procedures comply with U.S. federal and institutional standards for research involving minors. TN uses Firebase Authentication and Firestore only for secure, cloud-based storage of de-identified metadata (e.g., session timestamps and feature usage metrics). No personally identifiable information, such as names, contact details, school identifiers, or chat content, is stored or transmitted.

When the application interacts with external LLM services via API calls, no identifiable or user-generated text leaves the device. Simulation prompts are pre-constructed from standardized, non-personal templates to generate consistent, de-identified responses. No raw conversation data from coaches or student-athletes is transmitted to, stored by, or retained on any external LLM vendor’s servers.

All LLM API providers used for this study (e.g., Together API/Ollama) are contractually required to exclude all request and response data from model-training or retention pipelines. No data transmitted through these APIs is logged, cached, or stored beyond the brief period required for real-time response generation.

All communications between the app, Firebase, and LLM endpoints occur via encrypted HTTPS (TLS 1.3) connections. Data stored within Firebase are encrypted at rest (AES-256) and hosted on U.S.-based Google Cloud servers compliant with ISO 27001 standards (International Organization for Standardization [ISO], 2022). Access is restricted to authorized research personnel through institutional Google Workspace accounts with multi-factor authentication.

The study adheres to the Family Educational Rights and Privacy Act (FERPA) and the Children’s Online Privacy Protection Act (COPPA), ensuring that no education records or identifiable minor data are collected or disclosed. Because no raw conversational data are stored, retained, or transmitted, a Data Processing Agreement (DPA) with Firebase/Google and a Business Associate Agreement (BAA) or equivalent assurance from the LLM vendor confirms compliance with applicable data-protection standards.

A standardized escalation procedure (SOP) is in place for any participant who reports risk of harm or distress during the study. Suppose a student-athlete indicates suicidal ideation, self-harm, or significant emotional distress in any survey or communication. In that case, the research team will immediately notify the designated school counselor or mental-health lead, following the IRB-approved safety protocol. The counselor will then initiate appropriate follow-up in accordance with district policy. No automated system decisions are made by the app, and no identifiable data are transmitted to LLM vendors during this process.

No persistent data is retained beyond anonymized metadata (such as login information) required for system functionality. Participants may delete their account associated with their anonymized IDs at any time.

#### 2.7.4. Simulation Architecture, Latency, and Availability

TN uses a hybrid architecture. The mobile app performs on-device functions (UI, local state, lightweight redaction, and caching), while LLM inference for coach–athlete simulations is executed server-side via a managed API. Before any request is sent, the client strips potentially identifying tokens (names, school references, contact details) from coach inputs; no student-authored text is transmitted to LLM services. Requests are transmitted only in de-identified form and are not retained by vendors or used for model training.

Prior to any request being transmitted to external LLM APIs, TN applies a lightweight, deterministic client-side redaction layer designed to remove potentially identifying tokens from coach-authored inputs. This process uses a rule-based filtering pipeline that detects and strips common personally identifying patterns (e.g., names, email addresses, phone numbers, school identifiers, and location references) using regular expressions and fixed lexical rules before serialization of the request payload. Redaction is performed synchronously on-device, and only the sanitized text is eligible for transmission.

To ensure reliability, the redaction module was iteratively tested during development using synthetic inputs containing embedded identifiers to verify consistent removal prior to API calls. In addition, request payloads sent to LLM endpoints are logged locally in redacted form during development and staging environments to support auditing and verification that no identifying content is transmitted. While no automated system can guarantee perfect de-identification, this layered, client-side approach substantially reduces the risk of inadvertent disclosure and aligns with the platform’s design principle of minimizing data exposure by default.

### 2.8. Measures

Demographic characteristics and student-athlete outcomes will be measured using online surveys at the beginning, middle, and end of the season. Demographic characteristics include the ages, genders, races/ethnicities, and types of sports of coaches and student-athletes. Student-athletes will be asked to self-report their physical activity (over the past 7 days): “On how many days were you physically active for a total of at least 60 min per day? (Add up all the time you spent in any kind of physical activity that increases your heart rate or makes you breathe hard some of the time.)”

In addition, student-athletes will be asked to self-report four brief mental-health–related items adapted from validated adolescent screening tools. Items include the following:

(1) “When you feel sad or hopeless, are there adults that you can turn to for help?” (Centers for Disease Control and Prevention, 2023);
(2) “During the past two weeks, how often have you been bothered by feeling nervous, anxious, or on edge?” (Kroenke et al., 2007);
(3) “During the past two weeks, how often have you been bothered by not being able to stop or control worrying?” (Kroenke et al., 2007);
(4) “During the past 12 months, did you ever feel so sad or hopeless almost every day for two weeks or more in a row that you stopped doing some usual activities?” (Centers for Disease Control and Prevention, 2023).

These items are used descriptively to complement standardized scales and provide context for student-athlete well-being (Centers for Disease Control and Prevention, 2023; Kroenke et al., 2007).

#### 2.8.1. Sport Satisfaction

Student-athlete intrinsic satisfaction in sport participation will be measured through the Sport Satisfaction Instrument (SSI), which consists of two subscales (i.e., satisfaction/fun and boredom). Student-athletes will be asked to rate the extent to which they agree with each of the eight items, using a Likert scale from 1 (Strongly Disagree) to 5 (Strongly Agree). Studies have demonstrated reliability and validity (Granero-Gallegos et al., 2014; McCarthy et al., 2008).

#### 2.8.2. Sport Motivation

Based on the tenets of SDT, the Sport Motivation Scale-II is an 18-item instrument developed to measure the motivational level of student-athletes (Pelletier et al., 2013). SMS-II consists of six subscales, including amotivation, external regulation, introjected regulation, identified regulation, integrated regulation, and intrinsic motivation. Student-athletes will be asked to rate on a Likert scale the extent to which the motivations presented to practice sports align with their personal motives. Motivation was assessed using a 7-point Likert scale ranging from 1 (Do Not Agree at All) to 7 (Totally Agree). SMS-II is a valid measure across different studies and socio-cultural contexts (Pereira et al., 2024).

#### 2.8.3. Mental Well-Being

The 14-item Warwick–Edinburgh Mental Well-being Scale (WEMWBS) will be used to measure the different aspects of positive mental health of student-athletes (Tennant et al., 2007). The WEMWBS covers both hedonic and eudaimonic aspects of mental health, including positive affect, satisfying interpersonal relationships, and positive functioning. Student-athletes will be asked to best describe their experience of each statement over the past two weeks using a 5-point Likert scale from 1 (None of the Time) to 5 (All of the Time). The validation process is described in Tennant and colleagues (Tennant et al., 2007).

#### 2.8.4. Burnout

Student-athlete burnout will be assessed by the 15-item Athlete Burnout Questionnaire (ABQ), developed by Raedeke and Smith (Raedeke & Smith, 2001). Items are answered on a 5-point Likert scale. Student-athletes will be prompted to reflect on their current sport experiences and rate how often they experienced the feelings identified in each item, using a 5-point Likert scale from 1 (Almost Never) to 5 (Almost Always). The ABQ includes three subscales related to emotional/physical exhaustion, sport devaluation, and reduced sense of accomplishment. The ABQ is supported by various forms of evidence for its validity (Grugan et al., 2024).

#### 2.8.5. Coach–Athlete Relationship

The 11-item Coach–Athlete Relationship Questionnaire (CART-Q) will be employed to assess the multidimensional nature of the coach–athlete relationship (Jowett, 2009). The CART-Q consists of three subscales: commitment, closeness, and complementarity. CART-Q items will be measured on a 7-point scale ranging from 1 (Strongly Disagree) to 7 (Strongly Agree). The CART-Q has been widely used in youth sport studies with sufficient evidence to support its validity and reliability (Jowett, 2009; Woolliams et al., 2021).

#### 2.8.6. Autonomy-Supportive Coaching

Seven items relating to the student-athlete’s perceived autonomy-supportive coaching behaviors will be adopted from a previous study (Danitz et al., 2019). Student-athletes will be prompted to recall their sport experiences, with a specific focus on autonomy-supportive coaching behaviors during the season, and rate each item from 1 (Strongly Disagree) to 5 (Strongly Agree). Psychometric Instruments are illustrated in Appendix A.

#### 2.8.7. User Interface and User Experience

We will conduct exploratory, semi-structured interviews with a purposive sample of coaches and student-athletes at the end of the season to gather usability and user-experience (UI/UX) feedback on the TN platform. An interview guide developed by the research team (see Appendix B) will be used.

### 2.9. Data Analysis

Analyses will use linear mixed-effects models (LMMs) estimated via REML to account for nesting (student-athletes within teams within schools) and to accommodate unbalanced repeated measures. Fixed effects will include condition (intervention vs. waitlist control), time (baseline, mid-, and end-season), and their interaction; random intercepts will be specified for team and school. Missing data will be handled by maximum-likelihood under MAR; if attrition exceeds 20%, multiple imputation will be used as a sensitivity analysis. Prespecified covariates include type of sport, gender, grade, and prior mental-health support. Given the pilot nature and very limited clusters, results will emphasize descriptive effect sizes with 95% CIs and ICCs/design effects rather than confirmatory *p*-values; where *p*-values are reported, we will control the false discovery rate (Benjamini–Hochberg) across outcomes.

Because cluster-randomized designs with few clusters are susceptible to chance baseline imbalances, baseline values of each outcome will be included as covariates in all longitudinal models. This baseline-adjusted specification is expected to improve precision and reduce bias attributable to baseline differences between conditions. As a sensitivity analysis, estimates will be examined with and without adjustment for multiple comparisons to evaluate the robustness of observed patterns. These analyses are intended to support transparent interpretation of exploratory findings.

Verbatim transcriptions of coach and student-athlete interviews will be analyzed using rapid content analysis to identify key usability insights, perceived benefits, and improvement opportunities related to the TN platform. The analysis will prioritize efficiency and practical relevance in studies with small samples, maintaining analytic rigor through structured coding and documentation. Two researchers will independently review transcripts, generate preliminary codes, and summarize key points within a shared matrix. Codes will then be grouped into higher-order categories to capture salient patterns in user experience and acceptability (Danitz et al., 2019). Findings will be summarized thematically but interpreted as exploratory, descriptive feedback rather than claims of thematic saturation. NVivo 12 (QSR International, 2018) will be used for data organization and coding.

Trustworthiness will be addressed through multiple strategies: credibility will be strengthened by peer debriefing within the research team and participant validation of summary findings; dependability will be supported through an audit trail documenting analytic decisions; and confirmability will be ensured through a transparent, data-grounded coding process. Thick description of the study context and participant characteristics will promote transferability for similar school-based interventions.

## 3. Limitations

As a pilot feasibility study with a small number of clusters, this investigation has several important limitations that shape how its findings should be interpreted. Most notably, all quantitative inferences are intended to be interpreted as exploratory assessments of outcome trends rather than as confirmatory evidence of intervention effects. This study has several limitations that should be acknowledged. First, the sample size is modest and limited to two high schools. It limits the external validity of the findings to student-athletes from other geographic locations, educational environment, socioeconomic contexts, or athletic settings. With only two clusters, any between-arm statistical inference is fragile and at risk of inflated Type I error if clustering is ignored; our LMM framework and emphasis on estimation mitigate (but cannot eliminate) this concern. Second, randomization occurs at the team level within each school, which reduces contamination between intervention and control groups but also limits the number of independent clusters. This may reduce statistical power and increase the potential influence of team-level dynamics. Third, the reliance on self-report questionnaires introduces the possibility of response bias, particularly in domains such as stress and well-being, where student-athletes may under- or over-report depending on social desirability. While anonymity will be emphasized, the school-based setting may still affect the candor of responses. Furthermore, adherence is expected to vary, resulting in differing exposure that may attenuate group-level effects. Engagement with TN depends on having reliable smartphone access, data connectivity, and a willingness to use the app throughout the season. In under-resourced communities and populations, limited device availability, shared phones, or constrained data plans may reduce access and engagement, and introduce selection bias. To address this limitation, intervention usage will be objectively monitored via backend usage logs (e.g., logins, completed simulations, time per session, and engagement frequency across the season). Qualitative interview data from coaches and student-athletes will further contextualize usage variability by identifying perceived barriers, facilitators, and reasons for (dis)engagement. Polit data will support the design of future trials.

## 4. Expected Results

We expect to observe trends consistent with potential benefits of TN (e.g., improved satisfaction, motivation, and well-being; reduced burnout), alongside feasibility metrics (adherence, engagement, data completeness). Estimated ICCs and design effects will guide sample-size planning for a fully powered cluster-randomized trial.

## 5. Conclusions

Mental health is a growing concern, and many youth athletes in sport experience anxiety, depressive symptoms, burnout, and elevated risk of dropout. To the best of our knowledge, TN is the first AI-augmented mobile platform designed specifically to address these challenges in youth sport. The platform supports coaches in monitoring indicators of athlete well-being and in providing timely, supportive responses. Core features include simulated conversations for practicing communication skills and guided prompts for connecting athletes to appropriate school or community resources when needed. TN is lightweight and scalable, making it accessible to implement in diverse sports settings. It is coach-centered, designed to fit existing team routines with minimal burden, yet informed by athlete perspectives so that coaches can tailor support while preserving youth voice. Meanwhile, this approach reflects the primary role coaches hold in youth sport as key mediators of the motivational climate, gatekeepers of safety, and trusted adults to whom emerging concerns can be directed. TN is intended to be transferable to other organized sport programs and is available at no cost to coaches and athletes. We hope that the study results will clarify the effectiveness, feasibility, and implementation considerations, thereby supporting the scale-up and adaptation of this approach across various settings. Findings will also inform refinement of the application and best practices for digital mental health supports in youth sport.

## Data Availability

No new data were created or analyzed in this study.

## Appendix A. Psychometric Instruments

*Sport Satisfaction Instrument (SSI)*

I usually have fun doing sports.
I usually enjoy playing sports.
I usually get involved when I am doing sports.
I usually find time flies when I am doing sports.
I usually find playing sports interesting.
In sports, I often daydream instead of thinking about what I’m doing.
When playing sports, I am usually bored.
When I practice sports, I wish the competition were over soon.

*Sport Motivation Scale (SMS-II)*

Because I would feel bad about myself if I did not take the time to do it.
I used to have good reasons for doing sports, but now I am asking myself if I should continue.
Because it is very interesting to learn how I can improve.
Because practicing sports reflects the essence of whom I am.
Because people I care about would be upset with me if I didn’t.
Because I found it is a good way to develop aspects of myself that I value.
Because I would not feel worthwhile if I did not.
Because I think others would disapprove of me if I did not.
Because I find it enjoyable to discover new performance strategies.
I don’t know anymore; I have the impression that I am incapable of succeeding in this sport.
Because participating in sport is an integral part of my life.
Because I have chosen this sport as a way to develop myself.
It is not clear to me anymore; I don’t really think my place is in sport.
Because through sport, I am living in line with my deepest principles.
Because people around me reward me when I do.
Because I feel better about myself when I do.
Because it gives me pleasure to learn more about my sport.
Because it is one of the best ways I have chosen to develop other aspects of myself.

*The Warwick–Edinburgh Mental Well-being Scale (WEMWBS)*

I’ve been feeling optimistic about the future
I’ve been feeling useful
I’ve been feeling relaxed
I’ve been feeling interested in other people
I’ve had energy to spare
I’ve been dealing with problems well
I’ve been thinking clearly
I’ve been feeling good about myself
I’ve been feeling close to other people
I’ve been feeling confident
I’ve been able to make up my own mind about things
I’ve been feeling loved
I’ve been interested in new things
I’ve been feeling cheerful

*Athlete Burnout Questionnaire (ABQ)*

I’m accomplishing many worthwhile things in [sport]
I feel so tired from my training that I have trouble finding energy to do other things
The effort I spend in [sport] would be better spent doing other things
I feel overly tired from my [sport] participation
I am not achieving much in [sport]
I don’t care as much about my [sport] performance as I used to
I am not performing up to my ability in [sport]
I feel “wiped out” from [sport]
I’m not into [sport] like I used to be
I feel physically worn out from [sport]
I feel less concerned about being successful in [sport] than I used to
I am exhausted by the mental and physical demands of [sport]
It seems that no matter what I do, I don’t perform as well as I should
I feel successful at [sport]
I have negative feelings toward [sport]

*Coach–Athlete Relationship Questionnaire (CART-Q)*

I feel close to my athlete/coach.
I feel committed to my athlete/coach.
I feel that my sport career is promising with my athlete/coach.
I like my athlete/coach.
I trust my athlete/coach.
I respect my athlete/coach.
I feel appreciation for the sacrifices my athlete/coach has experienced in order to improve his/her performance.
When I coach my athlete/When I am coached by my coach, I feel at ease.
When I coach my athlete/When I am coached by my coach, I feel responsive to his/her efforts.
When I coach my athlete/When I am coached by my coach, I am ready to do my best.
When I coach my athlete/When I am coached by my coach, I adopt a friendly stance.

*Autonomy Supportive Coaching*

My coach gives us choices within specific rules and limits.
My coach explains why tasks and rules are important.
My coach lets us take initiative and work on our own.
My coach makes activities meaningful and challenging to motivate us.
My coach gives feedback that helps us feel capable without feeling controlled.
My coach praises behaviors that are within our control, focusing on how well we do them.
My coach gives each athlete individual attention, listens to our feelings and opinions, and helps us grow and develop.

## Appendix B. Interview Guide

*Coaches Interview Guide*

(1) When you open the dashboard, what draws your eye first? What do you look for next, and how have you used the coach dashboard this season?
(2) How did athlete check-ins and journals inform your coaching?
(3) How, if at all, have the simulated chats shaped what you say to athletes?
(4) In what ways do notifications fit or not fit your weekly workflow?
(5) Walk me through a moment when ThriveNudge changed a coaching decision or interaction.
(6) What would increase your trust in the app and make it more useful for you and your athletes?
(7) Recall a moment when the screen layout helped or hindered you during practice or right after a game.
(8) Which labels, icons, or features were unclear? How would you rename or reorganize them to match your coaching language and workflow?
(9) Which feature was most useful and which was least useful for you, and why?
(10) What specific changes to the interface (e.g., layout, navigation, wording, visualizations) would most improve your experience with ThriveNudge and better support your coaching?
(11) Is there anything I did not ask that you want to talk about?

*Student-athlete Interview Guide*

(1) Walk me through a typical check-in or journal you completed this season, when you did it, and the steps you took.
(2) Think back to a time your coach followed up after seeing your check-in. What happened, and how did it feel?
(3) Recall a moment when the app supported your motivation, mood, or connection with your coach. What made it helpful?
(4) What would increase your trust in the app, protect your privacy, and make you want to use it more often?
(5) When you start a check-in, what stands out on the screen, and what helps you finish?
(6) Describe a time the app felt confusing. Where did you get stuck or feel unsure about what to tap next?
(7) Which words, questions, or icons did not match how you talk about your season? How would you say or show them instead?
(8) Where do you change what gets shared with your coach? What would make those privacy/sharing settings clearer?
(9) Is there anything I did not ask that you want to talk about?
